# Zinc Finger Domain of the PRDM9 Gene on Chromosome 1 Exhibits High Diversity in Ruminants but Its Paralog PRDM7 Contains Multiple Disruptive Mutations

**DOI:** 10.1371/journal.pone.0156159

**Published:** 2016-05-20

**Authors:** Sonika Ahlawat, Priyanka Sharma, Rekha Sharma, Reena Arora, Sachinandan De

**Affiliations:** 1 National Bureau of Animal Genetic Resources, Karnal, India; 2 National Dairy Research Institute, Karnal, India; Chang Gung University, TAIWAN

## Abstract

PRDM9 is the sole hybrid sterility gene identified so far in vertebrates. PRDM9 gene encodes a protein with an immensely variable zinc-finger (ZF) domain that determines the site of meiotic recombination hotspots genome-wide. In this study, the terminal ZF domain of PRDM9 on bovine chromosome 1 and its paralog on chromosome 22 were characterized in 225 samples from five ruminant species (cattle, yak, mithun, sheep and goat). We found extraordinary variation in the number of PRDM9 zinc fingers (6 to 12). We sequenced PRDM9 ZF encoding region from 15 individuals (carrying the same ZF number in both copies) and found 43 different ZF domain sequences. Ruminant zinc fingers of PRDM9 were found to be diversifying under positive selection and concerted evolution, specifically at positions involved in defining their DNA-binding specificity, consistent with the reports from other vertebrates such as mice, humans, equids and chimpanzees. ZF-encoding regions of the PRDM7, a paralog of PRDM9 on bovine chromosome 22 and on unknown chromosomes in other studied species were found to contain 84 base repeat units as in PRDM9, but there were multiple disruptive mutations after the first repeat unit. The diversity of the ZFs suggests that PRDM9 may activate recombination hotspots that are largely unique to each ruminant species.

## Introduction

Genetic recombination during meiosis governs genetic variability of a species by shuffling of alleles between genes linked on the same chromosome. This confers each species the ability to withstand the pressure of natural selection [1]. Studies on pedigree, linkage disequilibrium and sperm typing have decrypted that recombination events occur in discrete 1–2 kb regions called “hotspots” punctuated by large cold domains, rarely involved in crossing over [2, 3]. How this non-random distribution of hotspots is achieved remained a mystery for quite some time. In the last few years, simultaneous work from many research groups has highlighted the importance of PRDM9 in specifying the location of meiotic recombination in humans and mice [4, 5, 6]

PRDM9 has three functional domains, an N terminal KRAB domain (promotes protein-protein binding), a central PR/SET domain (histone methyl transferase activity) and a terminal zinc finger (ZF) domain of cysteine2 histidine2 type [6]. Binding of PRDM9 to appropriate DNA sequences is mediated by its C2H2 zinc finger array. By virtue of its histone H3 lysine 4 methyltransferase activity, PRDM9 generates activated chromatin and guides the generation of double-strand breaks (DSBs) at these sites by SPO11 (topoisomerase-like protein) [7]. The revelation of gametogenesis arrest in meiotic prophase I and impaired double-strand break repair in PRDM9-null mice has further highlighted the importance of this gene [8].

The most fascinating feature of PRDM9 is the zinc finger (ZF) domain which has a minisatellite-like genomic structure and is encoded within a single exon. Each zinc finger (84 bp or 28 amino acids long) is repeated in tandem at both the DNA and protein levels with almost perfect homology [4]. Each sequential zinc finger binds a sequential trinucleotide on the target DNA molecule and thus influences recombination hotspot location [9].

There is a strong variability in the number of encoded zinc fingers in PRDM9 not only across species but also within species. The variation in PRDM9 zinc fingers can thus alter the location of genomic recombination hotspots [4]. The recombination hotspots are rarely conserved even between closely related species such as humans and chimpanzees despite over 99% genome sequence similarity [10]. It has also been established that PRDM9 is the most divergent of all human-chimpanzee pairs of orthologous zinc-finger proteins [5] and thus activates distinct hotspots in these two primate species during meiosis. Bovine genome encodes multiple paralogs of PRDM9 [11] and two independent studies have identified different PRDM9 paralogs to be associated with hotspot usage in cattle. Sandor et al. [12] observed that genome-wide hotspot usage is influenced by an X-linked PRDM9 paralog, but Ma et al. [11] identified paralog on chromosome 1 to be associated with recombination hotspot locations. The hotspot motifs undergo inevitable destruction during recombination, a phenomenon described as “hotspot conversion paradox” [13, 14]. A proposed resolution to this ‘paradox’ is that the variations in both number and sequence of ZF modify DNA binding specificities of PRDM9 at such a rapid pace that substantial depletion of any single recombination hotspot is prevented [4, 5].

Salient features of evolution of PRDM9 are that the zinc fingers are evolving under positive selection and concerted evolution across many species, particularly at positions involved DNA-binding [15]. The residues at positions -1, 2, 3 and 6 of each ZF determine the DNA-binding specificity of PRDM9, and are reported to be positively selected in humans [16], chimpanzees [17], equines [18] and mice [19, 20]. Interestingly, PRDM9 gene is absent in some taxa, such as chicken, frog and fruit fly and non-functional in others, such as opossum, nematodes and dog. Ray-finned fishes and tunicates possess PRDM9 but signals of positive selection and/or concerted evolution are lacking [15, 21]. These observations suggest that PRDM9 is not universally active in hotspot regulation and many taxa have perhaps evolved other mechanisms for specifying recombination hotspot locations [15]. In humans, PRDM9 gene has undergone duplication and the resulting paralog PRDM7 has experienced enormous rearrangements decreasing the number of encoded zinc fingers and altering the pattern of gene splicing [22]. PRDM7 has so far not been characterized in any other species except humans.

Intriguingly, PRDM9 has also been recognized as the only known mammalian speciation or hybrid sterility gene. Spermatogenic failure and resulting sterility of some *Mus m*. *domesticus* and *Mus m*.*musculus* hybrids has been attributed to allelic differences in PRDM9 [23]. PRDM9 is the fourth gene to be implicated in reproductive isolation after Odysseus-site homeobox, JYAlpha and Overdrive speciation genes which were identified in Drosophila [24, 25, 26].

Because of its important role in recombination and apparently noteworthy role in speciation, PRDM9 has become the focus of intense study in the last few years. Evolutionary dynamics of PRDM9 can only be unraveled by analyzing its allelic diversity in different species. Till date, diversity of PRDM9 has only been characterized in humans [5, 27], chimpanzees [17, 28], equines [18] and mice [19, 20]. Although the association of PRDM9 with meiotic recombination has been explored in cattle but there are no reports analyzing the diversity of this gene in other ruminant species. Hence, the aim of present study was, firstly, to characterize the zinc finger domain of PRDM9 paralog on chromosome 1 in small ruminants (*Ovis aries*, *Capra hircus*) as well as large ruminants (*Bos indicus*, *Bos grunniens*, *Bos frontalis*) and secondly, to explore the existence of PRDM7, a paralog of PRDM9 in these species.

## Materials and Methods

### Sample collection and DNA extraction

**Fig 1:**
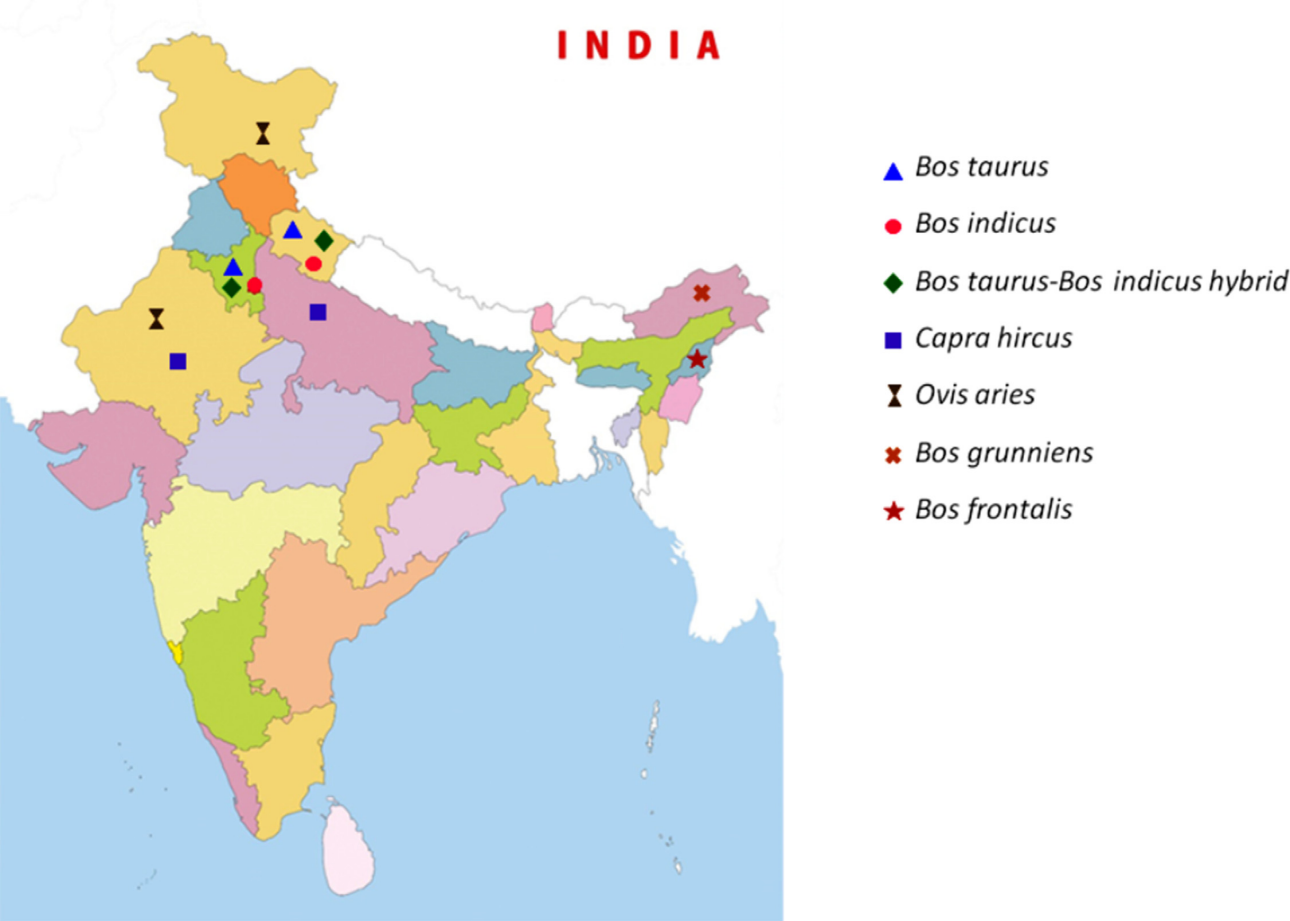
Geographic distribution of ruminant populations analyzed in the present study.

Animal experiments were not performed in this study; therefore, special approval from the ethics committee was not required. No specific permits were required for the sample collection, as none of the investigated species is an endangered or protected species. Blood samples were collected from two different states of India for cattle (Uttarakhand, Haryana), goat (Rajasthan, Uttar Pradesh) and sheep (Jammu and Kashmir, Rajasthan) and from one state each for Yak (Arunachal Pradesh) and Mithun (Nagaland). One hundred and five samples from cattle (*Bos taurus*, *Bos indicus*, *Bos taurus- Bos indicus* hybrid), 10 from yak (*Bos grunniens*), 20 from mithun (*Bos frontalis*), 45 from sheep (*Ovis aries*) and 45 from goats (*Capra hircus*) were collected specifically for this study with the help of veterinary doctors after obtaining permission from the respective State Animal Husbandry departments (Fig 1). Prior informed consent was obtained verbally from the owners of the animals in field. Genomic DNA was isolated from blood using phenol-chloroform method [29].

### PCR and sequencing

The zinc finger domain of PRDM9 paralog on chromosome 1 was amplified from genomic DNA of cattle, yak, mithun, sheep and goat. The primers for this study were designed manually using the *Bos taurus* PRDM9 sequence (KJ020105). Primer sequences used for amplification were: F- TGCTCTCTGGCCTTCTCCAGTCAGAA and R- GCTGCAGTAATTCTCCTGTGAC. PCR was carried out on an Veriti 96 well thermal cycler (Applied Biosystems) in 25 μL reaction mixture containing 2.5 μL of 10X Taq reaction buffer, 0.5 μl of 10mM dNTP mix (Fermentas), 0.5 μl of 10μM primer (each) and 0.25 units of Taq DNA polymerase (Sigma). The PCR reaction cycle was accomplished by denaturation for 5 min at 95°C; 30 cycles of denaturation at 94°C for 30 sec, annealing at 60°C for 30 sec, extension step at 72°C for 30 sec, with a final extension at 72°C for 10 min. PCR products were electrophoresed alongside DNA molecular weight marker in 1.5% agarose gel and visualized by staining with ethidium bromide. The amplified fragments were gel purified using PureLink Quick Gel Extraction Kit (Invitrogen Canada Inc.) and directly sequenced or subcloned into pTZ57R/T vector (Thermo scientific). Recombinant clones were selected and plasmid DNA was extracted. Multiple clones (2–3) were sequenced using Sanger sequencing to obtain the DNA sequence of the amplified products.

### Sequence analysis of ZF domains

The sequences were subjected to EBI- BLAT [30] (http://asia.ensembl.org/Multi/Tools/Blast?db=core) to perform pair-wise alignment with their corresponding genomes. The first gene hit with the highest score and lowest E-value was used to assign the name and identity of the sequence. The beginning and end of all zinc finger coding sequences were marked in the sequences by referring to chromosome location viewer of the ENSEMBL database. Accordingly, sequences were trimmed to remove ambiguities while maintaining the reading frame using BioEdit 7.0 Sequence Alignment Editor [31]. All the DNA sequences were translated using Translate tool of Sequence Manipulation Suite Version 2 [32]. Multiple sequence alignment of DNA and protein sequences of all species was performed using Clustal Omega with default parameters [33].

### dN/dS analysis of ZF domain coding sequences

The multiple sequence alignment of amino acid coding sequences of zinc finger domains in all species together and within each species depicted some sites to have high variability. Therefore, a Likelihood Ratio Test (LRT) was carried out to determine whether these sequences were subjected to positive selection. These test ratios were determined using software tool Codeml from user friendly interface PAMLX (Version PAML4.8a [34]. Nexus trees were constructed for ZF domain analysis using Mr. Bayes 3.2.2 software [35] based on Bayesian analysis by pre-setting amino acid model to mixed in order to find the best fit model and sample frequency set to 500. The analysis was run for 500000–600000 numbers of generations, till the average value of standard deviation of split frequencies decreased to below 0.05.

The input files for PAMLX consisted of DNA sequences coding for ZF domains aligned using CLUSTAL OMEGA in the standard sequential PHYLIP format and their corresponding tree constructed from Mr. Bayes. LRTs were obtained by performing codon based analysis by fixing branch lengths and alpha values. The site specific models compared for hypothesis testing included null model M1a (nearly neutral) vs M2a (selection) and M7 beta vs M8 (beta & ω) using F3X4 codon matrix with fixed alpha. Further, in case if positive selection was confirmed, Bayes Empirical Bayes approach was used to identify the specific sites exhibiting positive selection by referring to posterior probability values in the codeml output [36].

## Results

We amplified the final exon of PRDM9 paralog on chromosome 1 in five ruminant species (cattle-105, yak-10, mithun-20, sheep-45 and goats-45) using primers flanking the zinc finger domain region. The designed primers amplified genomic DNA unambiguously yielding homozygous or heterozygous ZF domain length variants of PRDM9 in the samples analyzed. An interesting observation was that a low molecular weight product of uniform size was constantly present across different samples of a species in addition to high molecular weight products of PRDM9 which varied within as well as between species. Intriguingly, all animals were homozygous for the low molecular weight product in all the species. Both high and low molecular weight products from representative homozygous samples of all the five ruminant species were sequence characterized after gel elution. The sequences of the high molecular weight product (around 1000 bp) from *Bos indicus* when aligned with *Bos taurus* genome assembly UMD3.1, indicated first hit to be to PRDM9 (ENSBTAG00000004538) located on chromosome 1 (98.35% identity) whereas only 2/3rd of the sequence could align perfectly with PRDM7 sequence present on chromosome 22 with 93.5% identity (S1 Fig). When the sequence of the low molecular product was subjected to alignment with the same genome assembly, the first hit was PRDM7 on chromosome 22 (99.04% identity). In contrast, only half of the query sequence aligned to PRDM9 on chromosome 1 with 96.5% identity. Similar observations were recorded for other species as well. The results for PRDM7 were confirmed by sub-cloning the gel purified product into pTZ57R/T vector (Thermo scientific) and 2–3 clones were sequenced using Sanger sequencing to obtain the DNA sequence.

**Fig 2:**
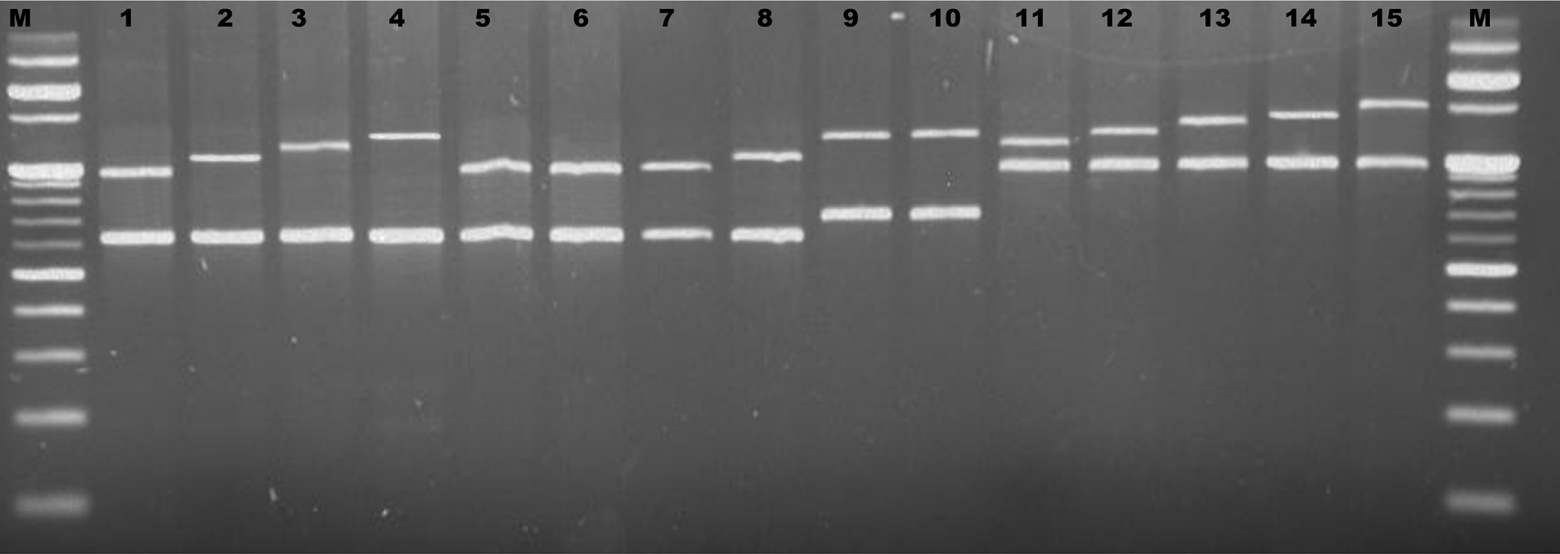
Gel image of PCR product of ZF domain of PRDM9 and PRDM7 genes in ruminants. M: 100 bp ladder, bright bands correspond to 500 bp, 1000 bp and 1500 bp. 1–4: upper band- A to D alleles of PRDM9 in cattle, lower band- PRDM7 in cattle. 5–6: upper band- A allele of PRDM9 in yak, lower band- PRDM7 in yak. 7–8: upper band- A and B alleles of PRDM9 in mithun, lower band- PRDM7 in mithun. 9–10: upper band- D allele of PRDM9 in sheep, lower band- PRDM7 in sheep. 11–15: upper band- C to G alleles of PRDM9 in goat, lower band- PRDM7 in goat.

In cattle, agarose gels resolved four different sizes of the PRDM9 ZF domain and these were named as A, B, C and D alleles of PRDM9. Sequence analysis revealed that alleles A, B, C and D contain 6, 7, 8 and 9 zinc fingers respectively. The size of PRDM9 allele in all the *Bos grunniens* (yak) samples corresponded to allele A of cattle PRDM9 which has 6 zinc fingers but in *Bos frontalis* (mithun), two alleles (A and B) with 6 and 7 zinc fingers were recorded. However, in all the *Bos* species (cattle, yak and mithun), ZF domain of PRDM7 was of the same size. Highest diversity of PRDM9 was observed in goats where five different PRDM9 alleles were observed and were designated as C, D, E, F and G with 8, 9, 10, 11 and 12 zinc fingers respectively. Various PRDM9 zinc finger domain length variants observed in different species are summarized in Tables 1 and 2. Interestingly, in most of the sheep samples analyzed, only one allele of PRDM9 (allele D) with 9 zinc fingers was produced, although a very small proportion of animals (8%) were heterozygous (CD). The size differences observed between PCR fragments for PRDM9 (Fig 2) were further compatible with variations in the number of copies of the 84 bp repeat unit since from allele A to G, there was sequential addition of one ZF repeat in the analyzed species. The size of ZF domain of PRDM7 was smallest in *Bos* species, intermediate in sheep and highest in goats.

**Table 1:** PRDM9 zinc finger domain length variants observed in large ruminants.

Species	Number of animals	AA	BB	CC	DD	AB	BC	AC
*Bos taurus*	25	1	10	1	-	2	10	1
*Bos indicus*	40	15	2	3	-	9	4	7
*Bos taurus- Bos indicus hybrid*	40	4	12	1	1	13	8	1
*Bos grunniens*	10	10	-	-	-	-	-	-
*Bos frontalis*	20	3	7	-	-	10	-	-

**Table 2:** PRDM9 zinc finger domain length variants observed in small ruminants.

Species	Number of animals	CC	DD	EE	FF	GG	CD	DE	EF	FG	CE	DF	EG	CF	DG	CG
*Capra hircus*	45	1	3	2	8	1	2	4	3	6	1	7	1	3	2	1
*Ovis aries*	45	-	41	-	-	-	4	-	-	-	-	-	-	-	-	-

We sequenced the final exon of PRDM9, which contains the ZF domains in 15 individuals (cattle-4, one each for the 4 alleles, yak-2, mithun-2, one each for the 2 alleles, sheep-2 and goats-5, one each for the 5 observed alleles). The size of these sequences ranged from 921 bp to 1326 bp for A to G alleles in the analyzed species. The sequences were submitted to GenBank and accessions numbers were obtained (KX109928-38, KU983505-06 and KU983508-09).

**Fig 3:**
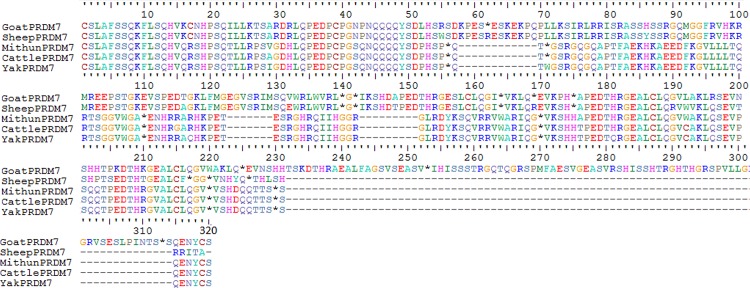
Alignment of PRDM7 ZF domains in ruminants. The stop codons are shown with stars.

One animal each of the five ruminant species was sequenced to obtain the sequence of the ZF domain of PRDM7 and the length of these sequences was 627 bp in cattle, yak and mithun, 710 bp in sheep and 960 bp in goats (KX109939-43). Zinc finger repeats (84 nucleotide stretches) were observed in all the species but *in silico* translation of PRDM7 sequence revealed presence of multiple stop codons. Interestingly, the location of the stop codons was more or less at the same sites in all the three *Bos* species (large ruminants). In small ruminants also, the location of these disruptive mutations was quite similar (Fig 3).

**Fig 4:**
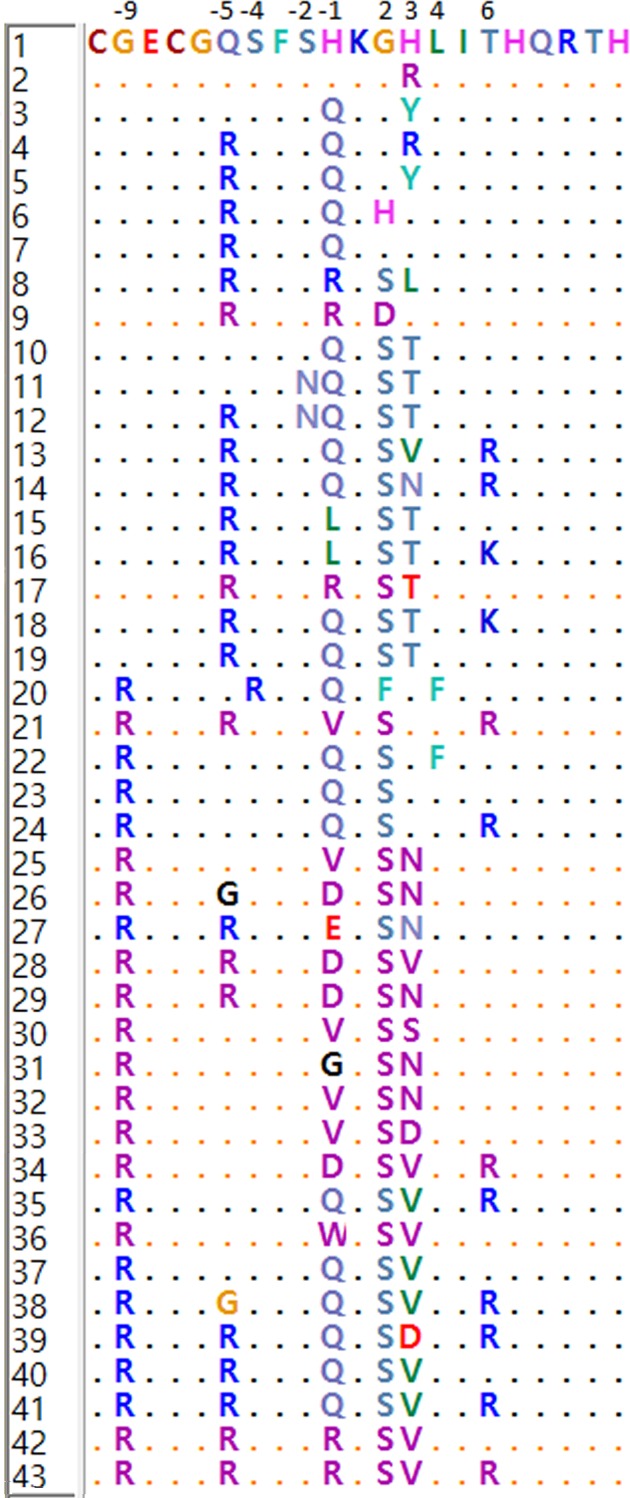
Different PRDM9 ZF domains identified in ruminant species. Different ZF domains have been numerically coded and amino acid variations at different positions have been indicated.

In all the species analyzed, the first finger (23 amino acid residues) was found to be highly conserved and was placed far apart from the minisatellite like domain structure which exhibited high variability. Ruminant PRDM9 sequences revealed presence of 43 different ZF domains characterized by amino acid variations at specific positions -9, -5, -2, -1, 2, 3 and 6 (Fig 4). In all the species, the final exon of PRDM9 comprised of a mixture of ZF domains. Some variants were common to specific taxa. For instance, amino acid glycine at position -9 was present in all large ruminants but arginine was seen in all small ruminants. Domains 1–19 were mainly seen in cattle, yak and mithun (large ruminants) whereas domains 20–43 were exclusively observed in sheep and goat (small ruminants). Some domains appeared on species-specific lineages. For instance, ZF domains 7, 8, 10 and 14 were identified only in yak, domains 6, 15 and 16 in mithun and domains 21, 25, 34, 42 and 43 in sheep. Nine domains were found to be unique in cattle and 18 were unique in goats. Identical PRDM9 ZF domain sequences were generally not shared between different species, with three exceptions: domain 18 was common in cattle and mithun, domain 19 in goat, cattle and mithun and finally domain 24 being shared between sheep and goat.

**Fig 5:**
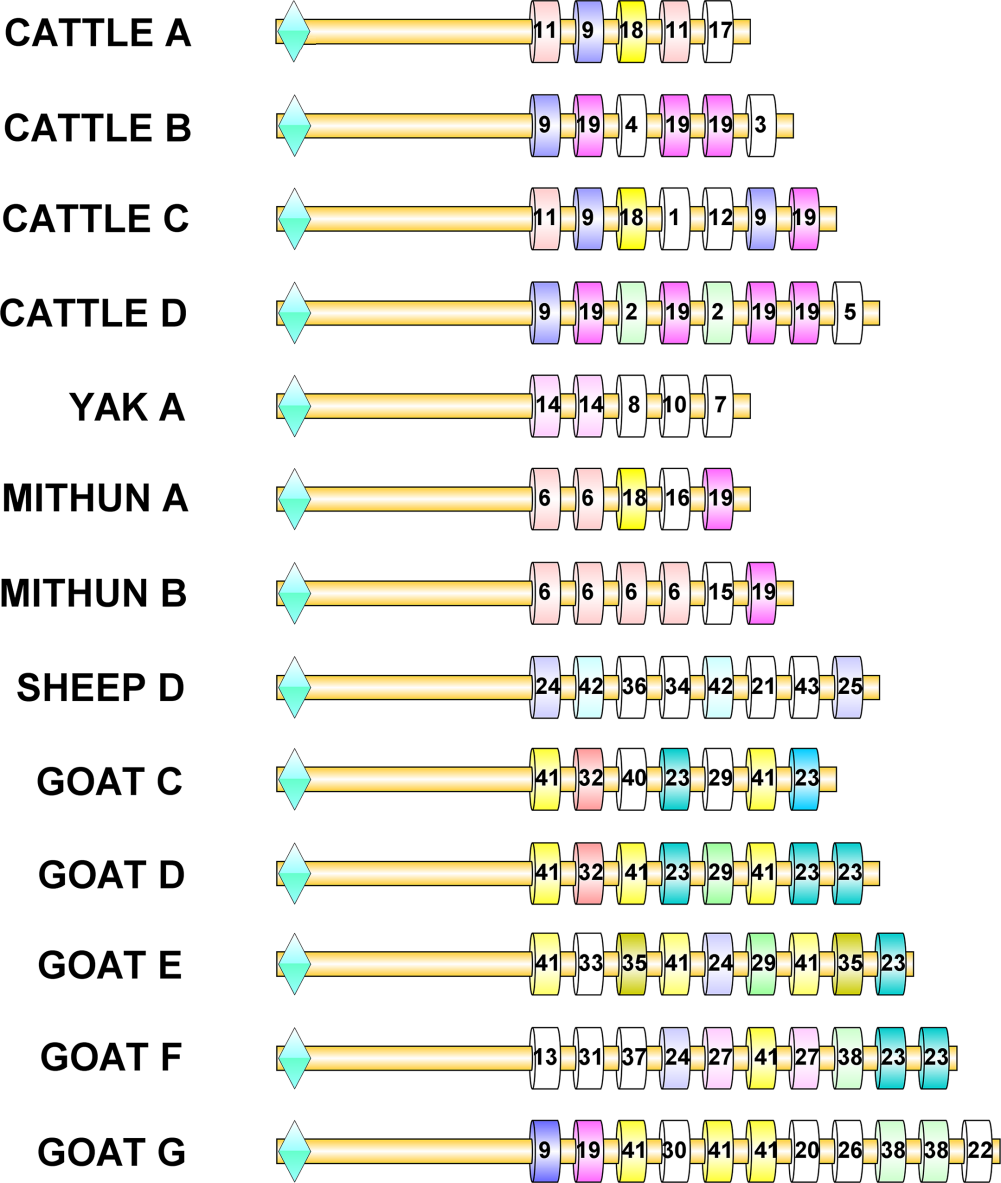
Schematic representation of PRDM9 domains and allelic variation in ruminants. Different ZF repeats in different alleles and species are coded by letters as already shown in Fig 4. The first finger (shown with green diamond shape) was found to be conserved.

Zinc finger array of some of the sequenced samples contained repeated ZF domains. Domain 6 was repeated in both A and B alleles of mithun, domain 19 was repeated in B and D alleles of cattle and domain 41 was common in all goat PRDM9 alleles (Fig 5). In particular, mithun showed the highest number of identical repeated domains. Interestingly, sheep contained the least number of repeated domains, with only domain 42 being repeated out of the 8 domains in the terminal zinc finger array.

Positions -1, 3, and 6 in the ZF domain of PRDM9 have been reported to show strong signals of positive selection in a number of species [15, 18, 19]. We examined whether the variation found in ruminant PRDM9 ZFs is consistent with the history of positive selection in other species. Evaluation of positive selection in ZF domains was done in each ruminant species separately and all species combined.

**Fig 6:**
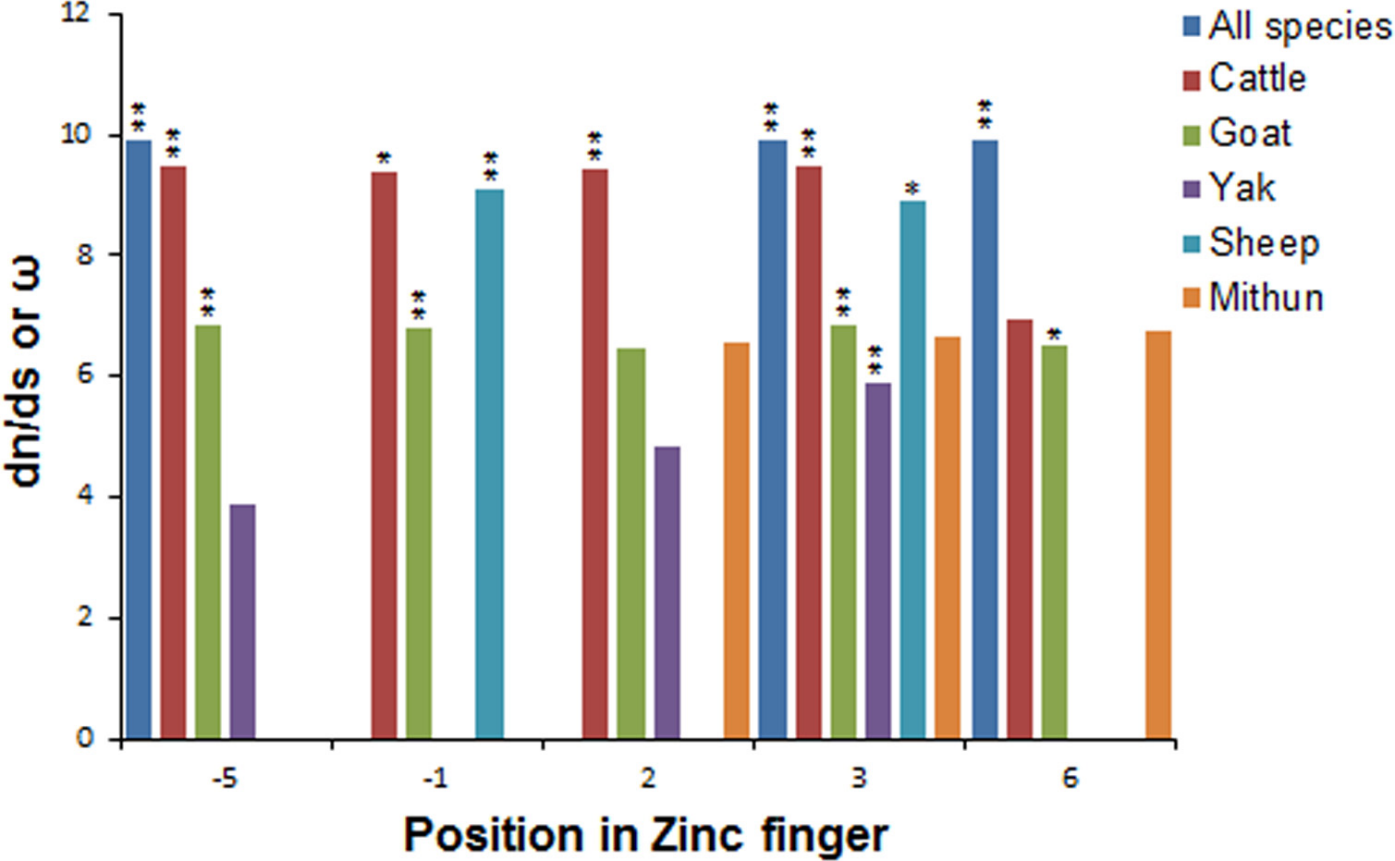
dN/dS estimates of variable amino acid sites in ZF domains of PRDM9 gene in ruminants. Positive selection was estimated in all species combined as well as for individual species separately. Two asterisks indicate P<0.01 and single asterisk indicates P<0.05.

When Zinc finger domain coding sequences were tested for selection by determining LRT using Codeml, highest LRT values were found in goat (28.84 for M2a vs M1a and 36.34 for M8(beta & ω) vs M7 model, both at 99% level of significance) and lowest LRT values (5.56 and 6.03) were obtained in mithun. Comparative analysis of site specific models (Table 3) yielded similar significant Chi-square p-values, when LRT of M1a vs M2a was compared with M7 and M8, except in case of yak and mithun, in which M1 vs M2 failed to show positive selection. However, considering significant results obtained from models M7 and M8 in all species, the sites of positive selection were further examined using BEB approach. The analysis indicated that position ‘3’ was universally positively selected in all species (Fig 6). The posterior mean of omega values ranged from as high as 9.47 (with high posterior probability) in cattle to as low as 6.7 in mithun (result not significant). Position ‘-5’ was found to be positively selected in all, except sheep and mithun, while yak and sheep did not show positive selection at position 6.

**Table 3:** The log likelihood ratio test (LRT) values to check for sites evolving under positive selection in zinc finger domains of different species.

Species	2Δl values for M2a vs M1a model	2Δl values for M8(beta & ω) vs M7 model
All species	214.3**	224.86**
Cattle	21.2**	21.3**
Yak	5.8	6.3*
Mithun	5.56	6.02*
Sheep	22.08**	22.48**
Goat	28.84**	36.304**

* indicates Chi-square test p-value < 0.05

** indicates Chi-square test p-value < 0.01

## Discussion

Homologous recombination during meiosis leads to reshuffling of both maternal and paternal alleles, thus contributing to genetic diversity [1]. Three simultaneous publications in Science identified PRDM9 as the gene responsible for specifying the location of recombination hotspots during meiosis in humans and mice [4, 5, 6]. Bovine genome is reported to possess multiple paralogs of PRDM9 [11]. Two separate studies have identified different PRDM9 paralogs to be associated with hotspot usage in cattle. Sandor et al. [12] characterized cattle male meiotic recombination in 10,192 Holstein Friesian (HF) bulls from the Netherlands and 3783 HF and Jersey bulls from New Zealand using 50K SNP chip. They observed that genome-wide hotspot usage is influenced by genetic variants in an X-linked PRDM9 paralog. A recent comprehensive study covering over half a million Holstein cattle with pedigree information by Ma et al. [11] reported recombination maps for both males and females by genome wide association study (GWAS) approach. Their analysis provided strong evidence that the PRDM9 paralog on chromosome 1 is associated with recombination hotspot usage.

In this study, we assessed the DNA sequence diversity in the zinc finger domain of PRDM9 on bovine chromosome 1 and its paralog, PRDM7 on chromosome 22 in five ruminant species. We found that although the sequenced region of PRDM7 contained 84 base repeat units characteristic of the PRDM family but there were multiple disruptive mutations after the first zinc finger. Horse PRDM7 has also been reported to contain early stop codons in the ZF domain [18]. In primates too, PRDM7 has undergone major structural rearrangements decreasing the number of encoded zinc fingers and modifying gene splicing [22]. In humans, the last exon coding for the Zn-fingers experienced partial deletion and the resulting protein was found to contain only 4 repeats instead of 14 repeats known for PRDM9. Study by Fumasoni et al. [22] reported that structural rearrangement of PRDM7 involved an 89-nucleotide long duplication in exon 3 within the sequence of the mature mRNA. This introduces a frameshift, and the resulting protein has modified C-terminal region. The duplication was confirmed in a variety of normal tissues as well as cancer cell lines indicating that this might be a general mechanism to produce an alternative PRDM7 protein without zinc fingers. Primate PRDM7 mRNA was found to be 2000 nucleotide long and coding for a protein with KRAB and PR domains and a C-terminal region of around 100 residues before encountering a stop codon. In our study also, stop codons in the ZF array sequence were seen after 165 residues in cattle, yak and mithun. The position of non-sense codons was after 183 residues in goat and 417 residues in sheep. In mouse also, two isoforms of the PRDM9 (*Meisetz)* gene are generated by alternative splicing and they too lack the zinc finger repeats and code for a protein with only the KRAB and the PR domains [8]. A recent study by Buard et al. [19] reported that the number of PRDM9 ZF repeats in several taxa of mice was 7 to 17. However, they observed that one sample out of 250 mice genotyped had only 2 repeats in the ZF array. The mouse harboring this shorter allele was heterozygous and the larger fragment had 9 ZF repeats. The shorter fragment with 2 ZFs was speculated to be a paralog of PRDM9 that is transcribed and translated in testis. Contrary to reports in other metazoans, no disruptive mutations were observed in this newly identified PRDM9 paralog in mice. There are few reports in vertebrates where duplicated genes use frameshift as a mechanism to diversify their function [37]. Therefore the frameshifted PRDM7 protein without zinc fingers could have novel roles to play in recombination. In case of dogs and other canids, PRDM9 coding sequence is disrupted because of multiple stop codons rendering it a pseudogene [38]. This reinforces the fact that multiple disruptive mutations are a common feature of evolution of these paralogs (PRDM9 and PRDM7) across different species.

PRDM9 ZF domains in ruminants showed remarkable numerical and amino acid composition variation. The number of ZF repeats varied from 6 to 12 and the number of different ZF domains was 43. Our results extend the spectrum of high variation of PRDM9 alleles reported in other organisms [15] suggesting rapid evolution of these domains. Sandor et al. [12] characterized the cattle PRDM9 paralog on chromosome X by designing primers that specifically amplify two adjacent gonosomal PRDM9 paralogs (PRDM9-XA and PRDM9-XB). The sequence of PRDM9-XA and PRDM9-XB ZF arrays was determined in 80 individuals and compared with PRDM9-XA and PRDM9-XB reference sequences (UMD3 build) which contain 8 and 20 ZF domains arranged in tandem, respectively. Sequence analysis revealed lack of polymorphism in the PRDM9-XA array. However, nine SNPs and a VNTR-like length polymorphism were observed in PRDM9-XB array, since a common allele with 22 ZFs could be detected. The number of ZF repeats in PRDM9-XA paralog is equal to the allele C of the present study, since both contain 8 zinc fingers. Interestingly, the zinc finger array of cattle PRDM9-XB is much longer than the PRDM9 paralog on chromosome 1 evaluated in our study. In humans, more than 40 PRDM9 alleles have been identified, each with a different DNA-binding specificity [4, 6, 39, 40]. The variance in genome-wide hotspot usage among human individuals has been attributed to PRDM9 allelic diversity [4]. Two different groups characterized 21 non-hominid ZFs across 25 alleles within the *Pan* genus [17, 28]. Recently, an additional 148 ZFs from 40 previously uncharacterized alleles were reported across 11 primate genera [41]. Similar work in 8 equid species found high variation in the number of ZF domains, ranging from 5 to 14 with 13 types of different ZF domains [18]. Diversity of PRDM9 zinc finger array in three sub-species of house mouse unraveled that the number of repeats extended from 7 to 17 and there were 78 different DNA alleles in the ZF domain [19]. Instability derived from the minisatellite structure of the ZF array has been suggested to be the cause of rapid evolution of PRDM9 ZF domains [21]. Recent study by Jeffreys et al. [42] reported that the rate of mutation (change of copy number and identity of zinc fingers) of the PRDM9 zinc finger array in humans was extremely high (at least 10^−5^ per generation). These observations support immense diversity of ZF domain of PRDM9 across diverse taxa.

In the different ZF domains, 9 among the 21 codons of the ZF unit showed nonsynonymous variability in our study. The most diverse codons were at positions -1, 2 and 3 of the ZF unit with nine, five and nine variant amino acids respectively. At position 6, there were three variant amino acids (T, R and K). Position -9 was the least diverse with two major variant amino acids (G and R) with G being specific for large ruminants and R being specific for small ruminants. Studies in multiple organisms have suggested that PRDM9 ZF domains evolve under strong positive selection [15, 16]. In our study, amino acid residues at positions -5, -1, 2, 3 and 6 were observed to bear positive selection in different species when analyzed separately as shown in Fig 6. However, analysis including all species together identified amino acids at positions -5, 3 and 6 to be positively selected in ZF domains of PRDM9 gene. The present study for the first time reports amino acid at position -5 to be positively evolving (p-value < 0.01 indicating 99% significant results) in the species analyzed. Signals of positive selection have also been associated with the PRDM9 paralog on chromosome 1 [11] and that on chromosome X [12] in cattle. In rodents and primates, divergent evolution due to positive selection at -1, 3, 6 positions which determine the DNA-binding specificity has been reported. In equids, positive selection was restricted to amino acid positions -1 and 6 [18] whereas in chimpanzees and bonobos, residues at positions -1, 3 and 6 were under positive selection [17]. Another study by Schwartz *et al*. [41] also reported positive selection at these positions in 11 primate genera. In case of wild mice, non-synonymous substitutions at these three amino-acid positions have been recently observed which suggest that natural selection is favoring such variations [19]. Therefore, our results are consistent with previous findings, which demonstrated positive selection on contact residues in other vertebrate species. It is worth emphasizing that the intra-specific variation in the ZF domains was observed to be less than the inter-species variation, which was again on expected lines. In particular, the highest number of identical repeated domains was seen in mithun and least in sheep. Greater sequence identity within species PRDM9 fingers is consistent with observations in other tandem satellite families, and is suggestive of concerted evolution of PRDM9 ZF array in ruminants.

The DNA-binding specificity of PRDM9 is determined by the residues at positions -1, 2, 3 and 6 of each ZF [43]. Since we observed remarkable diversity in the ZF at these positions, it suggests that PRDM9 may activate recombination hotspots that are largely unique to each ruminant species. These results are consistent with the lack of conservation in hotspot usage between chimpanzees and humans [28]. The introduction of new hotspots is imperative to counteract the loss of individual hotspots due to biased gene conversion upon double strand break repair. Evolution of both the PRDM9 protein and the hotspot motif offers a mechanistic solution to the “recombination hotspot paradox” [4, 5].

PRDM9 is the first (and to date only) locus associated with hybrid sterility in mammals [23]. An interesting example of reproductive isolation in bovines is the sterility of inter-species hybrids of cattle (*Bos taurus*) and yak (*Bos grunniens*) [44]. Pure yaks being poor milk and meat producers have been crossed with cattle to overcome poor production. The cattle-yak hybrid shows strong heterosis compared with cattle and yaks in terms of production performance such as meat, milk and draft power. However, reproductive isolation that results from the male sterility in the F1 hybrid is a major stumbling block to yak crossbreeding and exploitation of heterosis [45]. Lou et al. [46] explored the candidacy of PRDM9 on speciation in yak-cattle hybrids by evaluating the expression of PRDM9 in the testes of adult normal yaks, yak calves and hybrid sterile yaks. Their results showed that the mRNA levels of PRDM9 decreased dramatically in the testes of sexually immature yak calves and sterile male cattle-yaks compared with that of normal adult yaks. PRDM9 is expressed specifically in meiocytes after the animal attains puberty. This could be the reason for low expression of this protein in sexually immature yak calves. However, lower expression in sterile hybrids as compared to normal yaks suggests that PRDM9 gene might be associated with the male infertility of cattle-yaks. The present study reports that there is variation in the number and sequence of zinc fingers in PRDM9 gene between cattle and yaks. Therefore, activation of different recombination hotspots by virtue of different PRDM9 alleles in cattle and yak can be speculated to be one of the causes of hybrid sterility in the cattle-yak F1 male hybrids and may contribute to speciation in these bovines.

## Conclusion

The present study characterized PRDM9 on bovine chromosome 1 and its paralog PRDM7 in five ruminant species. Remarkable numerical and amino acid composition variation was observed in zinc finger domain of PRDM9 since 7 alleles with varying number of repeats (6–12) and 43 different ZF domains could be identified. Ruminant zinc fingers were found to be diversifying under positive selection and concerted evolution, specifically at positions involved in defining their DNA-binding specificity, consistent with the reports in other metazoans. PRDM7 in the studied species was found to contain multiple disruptive mutations, also reinforcing similar observations in humans and equids.

## Supporting Information

S1 Fig Snapshots of BLAT results of high and low molecular weight products. (TIF)
