# Supplementary figures and images for: Zinc Finger Domain of the PRDM9 Gene on Chromosome 1 Exhibits High Diversity in Ruminants but Its Paralog PRDM7 Contains Multiple Disruptive Mutations
Source: PLoS One. 2016 May 20;11(5):e0156159. doi: 10.1371/journal.pone.0156159 (PMC4874674; doi:10.1371/journal.pone.0156159)

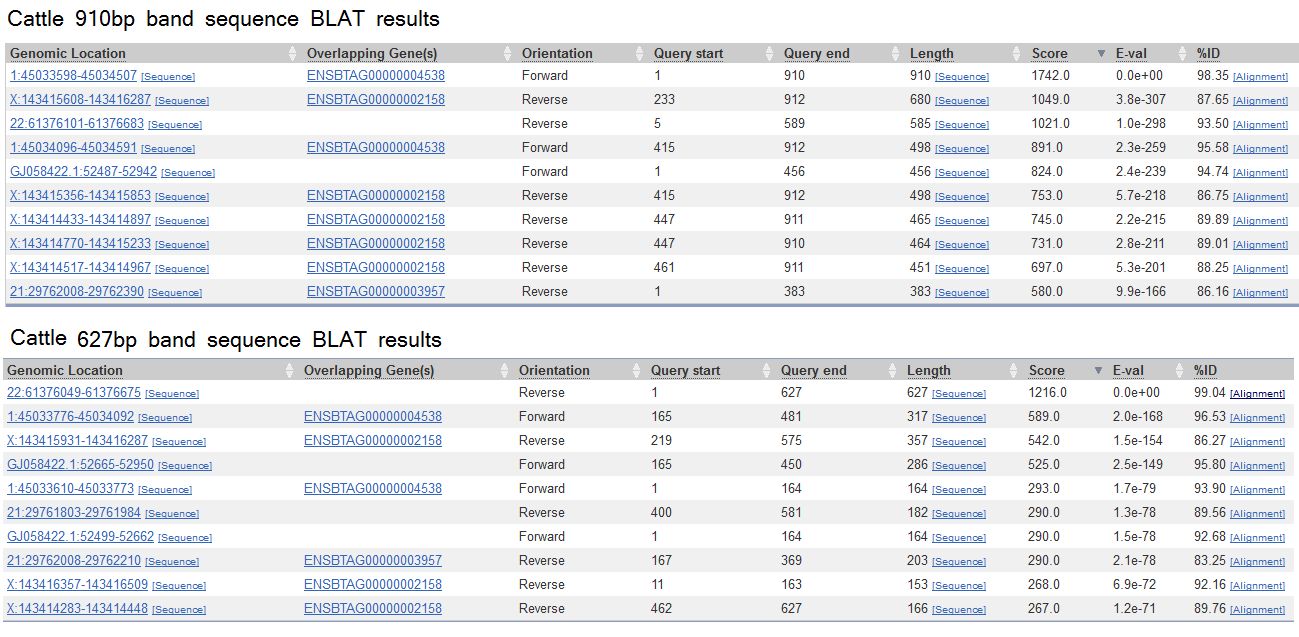

Supplement: S1 Fig — (TIF) [file pone.0156159.s001.tif]
